# Metabolomic Profiling of Poor Ovarian Response Identifies Potential Predictive Biomarkers

**DOI:** 10.3389/fendo.2021.774667

**Published:** 2021-11-23

**Authors:** Haixia Song, Qin Qin, Caixia Yuan, Hong Li, Fang Zhang, Lingling Fan

**Affiliations:** ^1^ Department of Reproductive Medicine, Shanxi Provincial People’s Hospital, Taiyuan, China; ^2^ Department of Central Laboratory, Shanxi Provincial People’s Hospital, Taiyuan, China

**Keywords:** poor ovarian response (POR), ovarian reserve, biomarkers, nicotinate and nicotinamide metabolism pathway, serum metabolomics

## Abstract

**Objective:**

To characterize the serum metabolomic profile and its role in the prediction of poor ovarian response (POR).

**Patient(s):**

Twenty-five women with normal ovarian reserve (24-33 years, antral follicle count [AFC] ≥5, anti-Müllerian hormone [AMH] ≥1.2 ng/ml) as the control group and another twenty-five women with POR (19-35 years, AFC <5, AMH < 1.2 ng/ml) as the study group were collected in our study. The serum levels of the women in both groups were determined from their whole blood by untargeted liquid chromatography–mass spectrometry (LC-MS). Multivariate statistical analysis and cell signal pathways analysis were used to reveal the results.

**Results:**

A total of 538 different metabolites were finally identified in the two groups. Tetracosanoic acid, 2-arachidonoylglycerol, lidocaine, cortexolone, prostaglandin H2,1-naphthylamine, 5-hydroxymethyl-2-furancarboxaldehyde, 2,4-dinitrophenol, and D-erythrulose1-phosphate in POR were significantly different from control as were most important metabolites in support vector machines (*p <*0.05). Metabolomic profiling, together with support vector machines and pathway analysis found that the nicotinate and nicotinamide metabolism pathway, including L-aspartic acid, 6-hydroxynicotinate, maleic acid, and succinic acid semialdehyde, was identified to have significant differences in POR women compared to control women, which may be associated with ovarian reserve.

**Conclusion:**

This study indicated that LC–MS-based untargeted metabolomics analysis of serum provided biological markers for women with POR. The nicotinate and nicotinamide metabolism pathway may offer new insight into the complementary prediction and therapeutic potential of POR. The functional associations of these metabolites need further investigation.

## Introduction

Poor ovarian response (POR), also called low ovarian response, is characterized by the pathological state of poor ovarian response to gonadotropin (GN) stimulation. At present, the diagnosis of POR is still unclear without unified criterion. Compared with Bologna standard (1), the Poseidon standard (2) wholly considers the age, ovarian reserve, and responsiveness of POR patients. This standard reduces population heterogeneity. In the process of ovulation induction of assisted reproductive technology (ART), some patients suffer from a low peak value of blood estrogen (E_2_) on hCG administration day and a low number of retrieved oocytes, which are often accompanied by the large consumption of GN and a high cycle cancellation rate, which are collectively known as low ovarian response (POR).

How to improve the pregnancy rate and pregnancy outcome of POR patients is the main aim and difficulty of reproductive research. Current main diagnostic indexes of ovary reserve include age, basal serum follicular stimulating hormone (FSH), basic serum inhibin B, serum anti-Müllerian hormone (AMH), antral follicle count (AFC), basal ovarian volume, basal serum E_2,_ and so on (3). The basal luteinizing hormone (LH)/FSH ratio and ovarian blood flow have also attracted attention (4, 5). A retrospective cohort study showed a novel mathematical model of true ovarian reserve assessment named the AAFA model based on predicted probability of POR: AMH, AFC, FSH, and age, in that order (6). However, how can we predict POR before these indicators change? We want to use metabolites in the serum as biomarkers in POR to infer whether these metabolites are related to ordinary diet or living environment, so as to provide more etiology guidance for the prevention or early treatment of POR.

Metabolomics is a global biochemical approach to test endogenous metabolites in biological systems and their dynamic changes in response to various factors (7–10). It has been widely used to predict and diagnose several diseases (11), especially in cancers to identify biomarkers in urine and serum among patients (12–14). Metabolomics has also been used to predict useful biomarkers in polycystic ovary syndrome (PCOS) (15–17).

There are few studies that identify biomarkers in serum in patients with POR by using metabolomics. We aim to characterize serum metabolomic profiling of POR patients using a liquid chromatography–mass spectrometry (LC-MS)-based untargeted metabolomics approach, and subsequently to identify the potential biomarkers and metabolic pathway for the predictive significance and therapeutic potential of POR.

## Materials and Methods

### Sample Collection

The clinical data of 50 women younger than 35 years old were collected from the Department of Reproductive Medicine of Shanxi Provincial People’s Hospital between January 2021 and May 2021. The study was approved by the Ethics Committee of Shanxi Provincial People’s Hospital. All the participants provided written informed consent. Their hormones were examined and they underwent transvaginal ultrasonography, to determine the level of basal FSH, LH, E_2_, progesterone (P), prolactin (PRL), testosterone (T), AMH, and AFC. According to different ovarian function status based on Poseidon Group 3, they were grouped into 25 women with normal ovarian reserve (as controls) and 25 women with POR.

The inclusion criteria for participants were as follows. Women aged 24-33 years, AFC ≥5, and AMH ≥1.2 ng/ml were included as the control group. Women aged 19-35 years, AFC <5, AMH <1.2 ng/ml were included as the study group (Poseidon Group 3).

Women with the following conditions including endometriosis, a history of oophorectomy, congenital adrenal hyperplasia, thyroid disease, hyperprolactinemia, and other diseases affecting ovarian reserve were excluded.

Venous blood samples (3 mL) were taken on days 2-4 of the menstrual cycle after spontaneous or withdraw of progesterone in the presence of amenorrhea to determine the levels of hormones, after an overnight fast of at least 10 h. AFC was calculated by counting the number of follicles in diameter with ranges from 2 to 9 mm in right and left ovaries by transvaginal ultrasonography. The plasma samples were isolated following centrifugation at 1,024 g for 10 min. All of the clinical characteristics were examined at the laboratory of the Department of Reproductive Medicine of Shanxi Provincial People’s Hospital. The samples were collected with a separating gel-promoting tube, and then centrifugated. The upper serums were stored at -80°C until use.

### Sample Preparation

All samples were centrifuged at 4°C for LC-MS analysis. A total of 100 μL of each sample and 400 μL of methanol (-20°C) were transferred and mixed into 2 mL centrifuge tubes. The mixture was vortexed for 60 s and centrifuged at 4°C for 10 min at 12000 rpm. The supernatant was transferred from each sample into another 2 mL centrifuge tube and was concentrated until dry in a vacuum. In total, 150 μL of 2-chlorobenzalanine (4 ppm) 80% methanol solution was added to dissolve samples, and the supernatant was filtered through a 0.22 μm membrane to obtain the prepared samples for LC-MS. A total of 20 μL of each sample was taken for quality control (QC).

### LC-MS Analysis

Chromatographic separation was accomplished in an Thermo Ultimate 3000 system equipped with an ACQUITY UPLC^®^ HSS T3 (150×2.1 mm, 1.8 μm, Waters) column maintained at 40°C. The temperature of the autosampler was 8°C. Gradient elution was carried out with 0.1% formic acid in water (C) and 0.1% formic acid in acetonitrile (D) or 5 mM ammonium formate in water (A) and acetonitrile (B) at a flow rate of 0.25 mL/min. Injection of 2 μL of each sample was done after equilibration. An increasing linear gradient of solvent B (v/v) was used as follows: 0~1 min, 2% B/D; 1~9 min, 2%~50% B/D; 9~12 min, 50%~98% B/D; 12~13.5 min, 98% B/D; 13.5~14 min, 98%~2% B/D; 14~20 min, 2% D-positive model (14~17 min, 2% B-negative model).

The ESI-MSn experiments were executed on the Thermo Q Exactive Focus mass spectrometer with a spray voltage of 3.5 kV and -2.5 kV in positive and negative modes, respectively. Sheath gas and auxiliary gas were set at 30 and 10 arbitrary units, respectively. The capillary temperature was 325°C. The orbitrap analyzer scanned over a mass range of m/z 81-1 000 for a full scan at a mass resolution of 70,000. Data-dependent acquisition (DDA) MS/MS experiments were performed with a HCD scan. The normalized collision energy was 30 eV. Dynamic exclusion was implemented to remove some unnecessary information in MS/MS spectra.

### Data Processing

Raw data files were processed by the Proteowizard software (v3.0.8789). The XCMS package (v3.1.3) was used for peak identification, peak filtering, and peak alignment. We obtained a data matrix including mass to charge ratio (m/z), retention time (RT), and peak area (intensity). A total of 14881 peak indices in positive mode and 11947 peak indices in negative mode were observed between the samples. The data were exported to Excel for subsequent MetaboAnalystR analysis (18–20).

### Metabolite Annotation and Pathway Analysis

We conducted quality control (QC) and standardization of sample data. Histogram, thermogram, and unsupervised dimension reduction analysis (principal component analysis, PCA) were used to obtain the statistics of metabolite content. Supervised partial least squares discriminant analysis (PLS-DA) and supervised orthogonal partial least squares discriminant analysis (OPLS-DA) were used to measure the quality and reliability of the model by R^2^Y and Q^2^. Univariate analysis and machine learning [random forest, support vector machines (SVM)] were used for screening the importance of biomarkers by the area under the curve (AUC). Pathway analysis of characteristic metabolites (enrichment analysis, topology analysis, etc.) could also be used for identifying important metabolic pathways.

### Statistical Analysis

The data of baseline clinical characteristics of all the subjects were analyzed by SPSS (version 23;SPSS Inc.). The Kolmogorov-Smirnov test (K-S test) was used to estimate normality of distribution. Normally distributed continuous variables were presented as means ± standard deviations (SDs) and Student’s t-test was used to compare the statistical difference between the two groups. Non-normally distributed numerical variables were presented by medians (25th to 75th percentiles) and the nonparametric test was used to compare the statistical difference between the two groups. Two-sided *p*-values <0.05 were considered statistically significant.

## Results

### Baseline Characteristics of POR Patients and Controls

A total of 50 subjects were enrolled in our study, including 25 control women with normal ovarian reserve (24-33 years, AFC ≥5, AMH ≥1.2 ng/ml) and 25 study women with POR (19-35 years, AFC <5, AMH <1.2 ng/ml) diagnosed by Poseidon Group 3. The clinical characteristics are shown in **Table 1**. The mean age in the study was 29.36 ± 3.42 years, varying from 19 to 35 years. There were no statistically significant differences in age, LH, E_2,_ and T between the two groups (*p*>0.05). Similar to another study, an increase in the levels of FSH but a decline in AMH and AFC in the POR group showed significant differences (*p*<0.001). The POR group had a significantly lower PRL than the control group (*p*<0.05), but all were in the normal range.

**Table 1:** Clinical characteristics of controls and POR group (median, 25^th^ -75^th^ ).

Variables	Control group (n = 25)	POR group (n = 25)	P value
Age (y)	29.00 (27.50-31.00)	31.00 (26.50-34.00)	0.287
FSH (miU/mL)	7.14 (6.49-7.53)	20.96 (12.00-26.10)	<0.001
LH (miU/mL)	3.65 (3.08-4.62)	4.30 (3.18-12.34)	0.200
Ez (pg/ml)	35.00 (27.50-39.41)	40.00 (28.07-71.15)	0.053
T (ng/dl)	35.08 (25.15-44.41)	29.00 (19.5-37.75)	0.233
PRL (ng/m1)	14.28 (11.89-18.56)	10.00 (8.98-14.64)	0.004
AMH (ng/ml)	4.34 (3.95-5.00)	0.07 (0.04-0.53)	<0.001
AFC (n)	18.00 (16.00-20.00)	4.00 (2.50-4.00)	<0.001

FSH, follicle stimulating hormone; LH, luteinizing hormone; E2, estradiol; T, testosterone; PRL,prolactin; AMH, antimmüllerian hormone; AFC, antral follicle count.

### Quality Assessment and QC of Serum Samples

**Figure 1:**
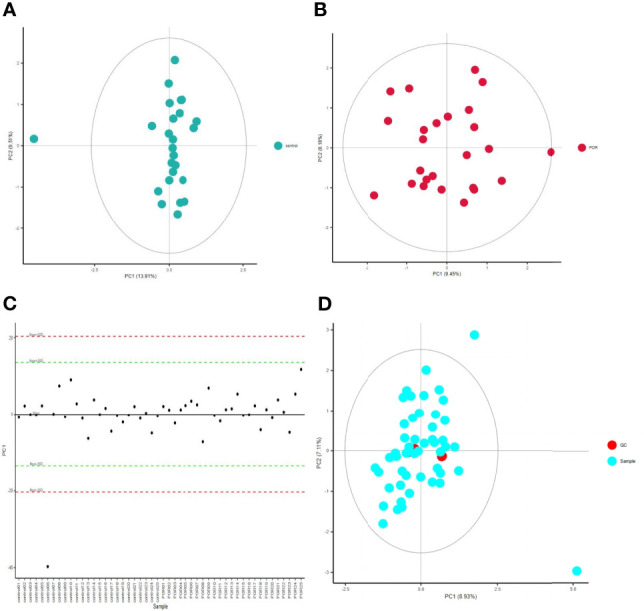
PCA score chart of control group **(A)** and POR group **(B)**, and distribution of PC1 in each sample point **(C)**, PCA chart of QC sample **(D)**. **(C)** The abscissa is the test sample, and the ordinate corresponds to the PC1 value of the sample in PCA analysis. **(D)** The red dot is the corrected quality control sample point (QC sample), and the blue dot is the test sample.

QC is one of the basic concepts of bioanalysis, which is used to ensure the repeatability and accuracy of data measured by omics. Using unsupervised PCA, we modeled the samples of each group, and then displayed the score plot (**Figures 1A, B**). This intra-group PCA analysis discarded the interference between groups, allowing us to more clearly observe the variation within the group and find possible outliers. Each point in the graph represents a sample, and the position of the point in the graph is determined by all metabolites in the sample. The ellipse in the figure is the 95% confidence interval calculated and drawn based on Hotelling’s T2 metric. The sample fell outside the ellipse, suggesting that this point might be an outlier. However, we usually only considered deleting a sample when there were serious outliers, such as pulling the whole model out of shape.

The distribution of PC1 values of all sample points was used to evaluate whether the sample preparation and measurement process was controllable, and most points would fluctuate up and down around the axis within 2 standard deviations above the mean (2SDs) (**Figure 1C**). In this process, the QC-RFSC algorithm was used to correct the characteristic signal peaks of each sample (each metabolite), and the correcting effect of each metabolite was recorded. After correcting the signal drift, the QC sample points were gathered together in the PCA diagram, which proved that the correcting effect was good (**Figure 1D**).

**Figure 2:**
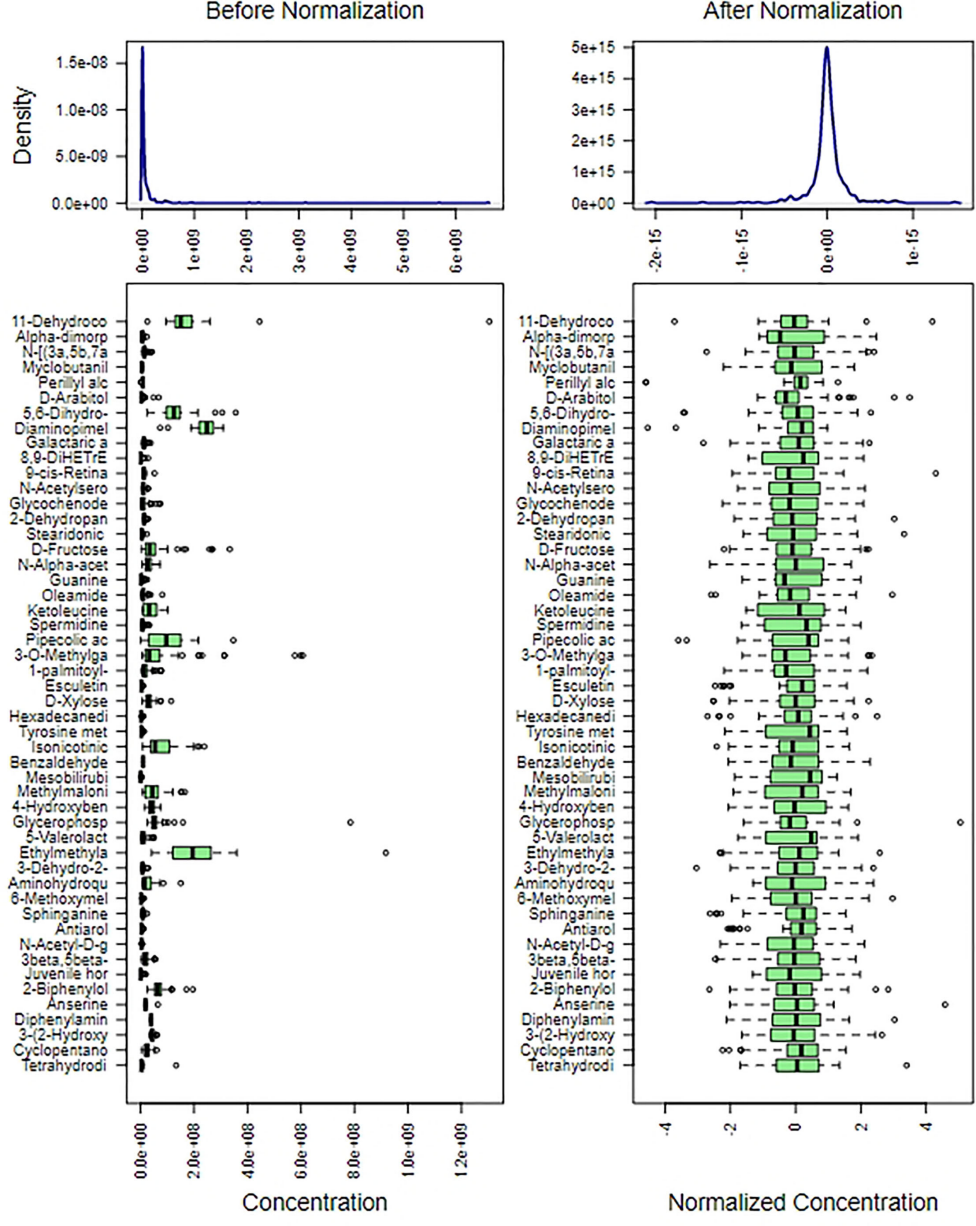
Content distribution of each metabolite before and after normalization. The content distribution was represented by box plot, which was corresponded to outlier, minimum, lower quartile, median, upper quartile, maximum and outlier from left to right. The figure on the left shows the distribution before normalization, and the figure on the right shows the distribution after normalization.

We used metabolite normalization for the purpose of making the mean and SD at the same level, and ensuring the later accuracy analysis of importance of all metabolites (**Figure 2**).

### Concentration Summary

**Figure 3:**
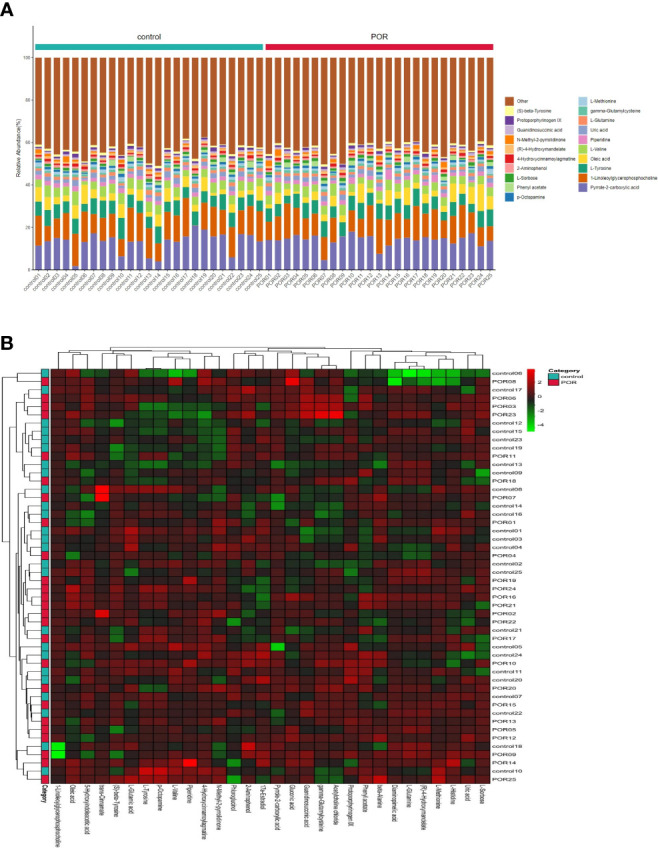
Accumulation histogram **(A)** and heat-map **(B)** of differential metabolites visualized the variations of metabolite profiles of POR and controls. **(A)** The abscissa is the sample name, which is sorted according to the grouping order, and different grouping samples are marked with different colors. The ordinate represents the percentage content of each metabolite, and the column order of corresponding metabolites from top to bottom is consistent with the legend. **(B)** The vertical axis is the sample name information, and also includes the grouping information. The horizontal axis is the metabolite. The cluster tree at the top of the figure is the similarity cluster of the distribution of metabolites in each sample, and the cluster tree at the left is the sample cluster tree, and the heat map in the middle is the heat map of metabolite content. The relationship between color and Z-score is shown in the scale at the top right.

Overall, 538 different metabolites were finally identified in the two groups. The content percentage of each metabolite in each sample was calculated and visualized by a stacking column diagram, which can intuitively compare the differences of metabolite composition and structure between groups. **Figure 3A** shows the top 20 content percentage of metabolites from low to high, including (S)−beta−tyrosine, protoporphyrinogen IX, guanidinosuccinic acid, N−methyl−2−pyrrolidinone, (R)−4−hydroxymandelate, 4−hydroxycinnamoylagmatine, 2−aminophenol, L−sorbose, phenylacetate, p−octopamine, L−methionine, gamma-glutamylcysteine, L−glutamine, uric acid, piperidine, L−valine, oleic acid, L−tyrosine, 1−linoleoylglycerophosphocholine, and pyrrole−2−carboxylic acid.

The clustering analysis of samples by heatmap found the similarity between different products, and we constructed the clustering model of samples. The samples of different groups were clustered to different positions. **Figure 3B** indicated that the composition and structure of the top 30 metabolites were quite different between groups.

### Supervised Partial Least Squares Discriminant Analysis

**Figure 4:**
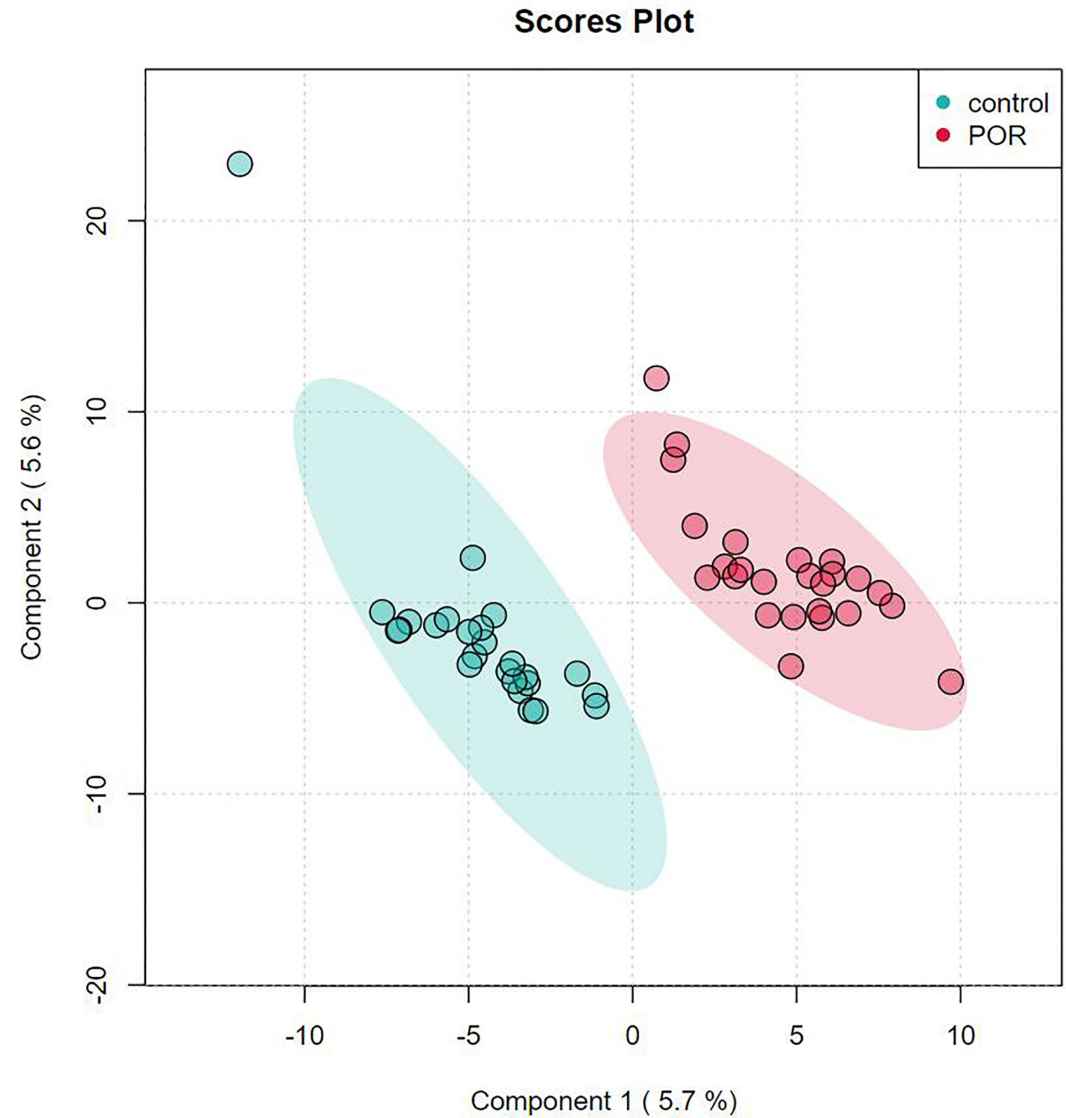
Score plot analysis of PLS-DA of group of POR and controls. Each point corresponds to a sample, and the abscissa and ordinate are the values of the two factors with the best discrimination effect. Different groups are marked with different colors, and the area marked by ellipse is the 95% confidence area of sample points.

PLS-DA is supervised, that is, grouping information needs to be provided during analysis. PLS-DA looks for factors that can distinguish the grouping of samples to the greatest extent (factors can be understood as the weighted sum of all metabolites). Discriminant analysis encodes the discontinuous classification variable to be predicted as a latent variable, and the latent variable is continuous. In this way, a regression can be established between the explanatory variable and the latent variable, which can be solved by the theory of least square regression. Partial least squares discriminant analysis is an upgraded version of linear discriminant analysis, which is suitable for omics data with a large number of collinearity problems in explanatory variables. PLS-DA finds a linear regression model by projecting the prediction variables and observation variables into a new space (each dimension of the new space is independent of each other and there is no collinearity problem). As shown in **Figure 4**, we used the two factors with the best discrimination effect to draw the scatter diagram. The point clouds of the two groups were obviously distributed in different areas, which indicated the good discrimination effect of the PLS-DA model and the distant differences in the composition and structure of metabolites of the two groups.

### Supervised Orthogonal Partial Least Squares Discriminant Analysis

**Figure 5:**
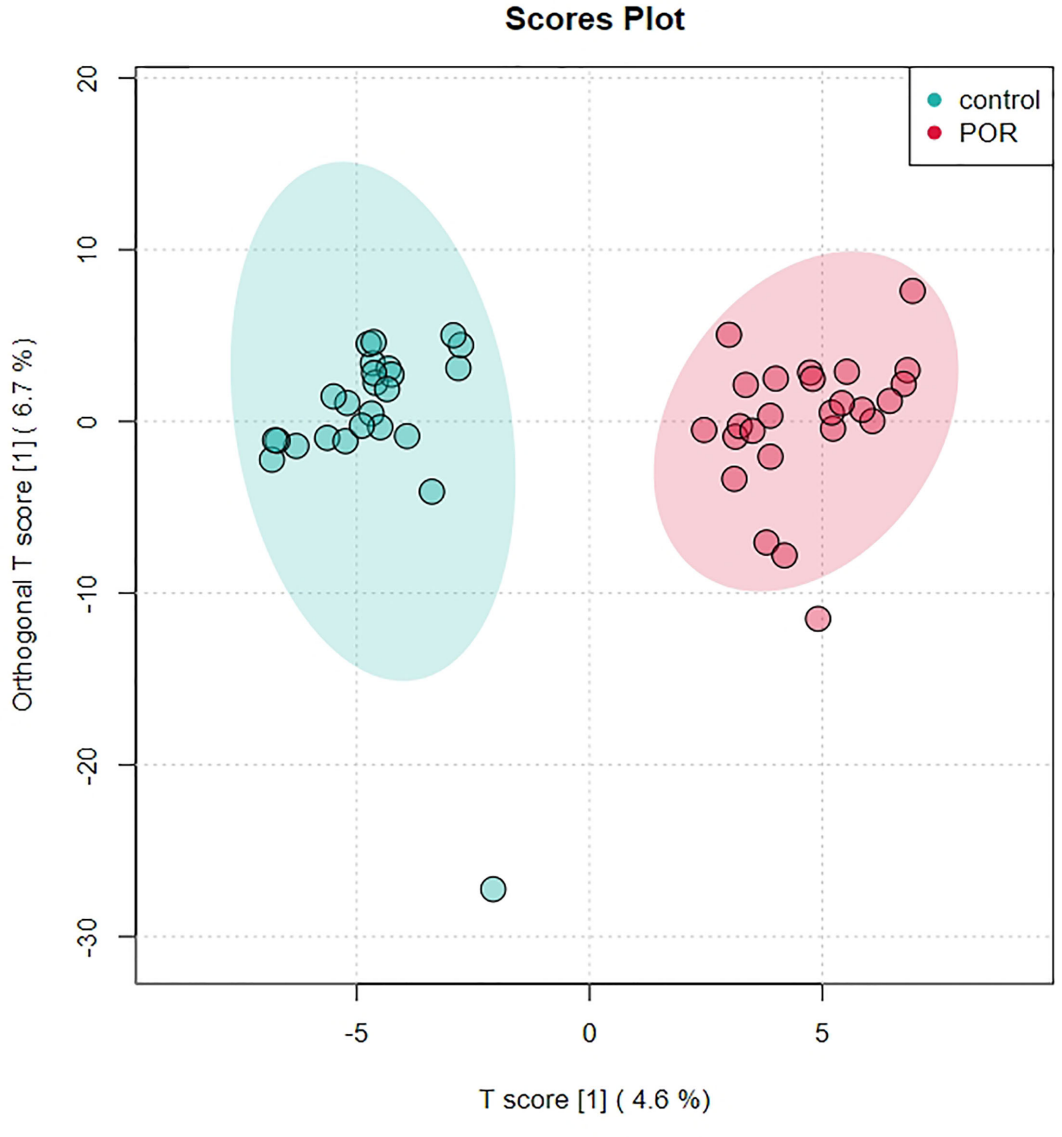
Score plot analysis of OPLS-DA of group of POR and controls. Each point corresponds to a sample. Different groups are marked with different colors, and the region marked by ellipse is the 95% confidence region of sample points.

Partial least squares discriminant analysis (PLS-DA) is also supervised, that is, grouping information is needed. OPLS-DA is an improvement of PLS-DA. The regression model is established between metabolome data and grouping information, and the model will filter out the information irrelevant to grouping. Compared with PLS-DA, the advantage of OPLS-DA is that only one component of metabolome data is used to predict grouping, while other components are used to describe orthogonal (unrelated) variation with the predicted component. In OPLS-DA, we usually use R^2^Y and Q^2^ to measure the quality and reliability of the model. The two values are usually close. The closer R^2^Y and Q^2^ are to 1, the better the discrimination effect of the model. The OPLS-DA model achieved better separation (R^2^X = 0.113, R^2^Y = 0.931, Q^2^ = 0.612; **Figure 5**).

**Figure 6:**
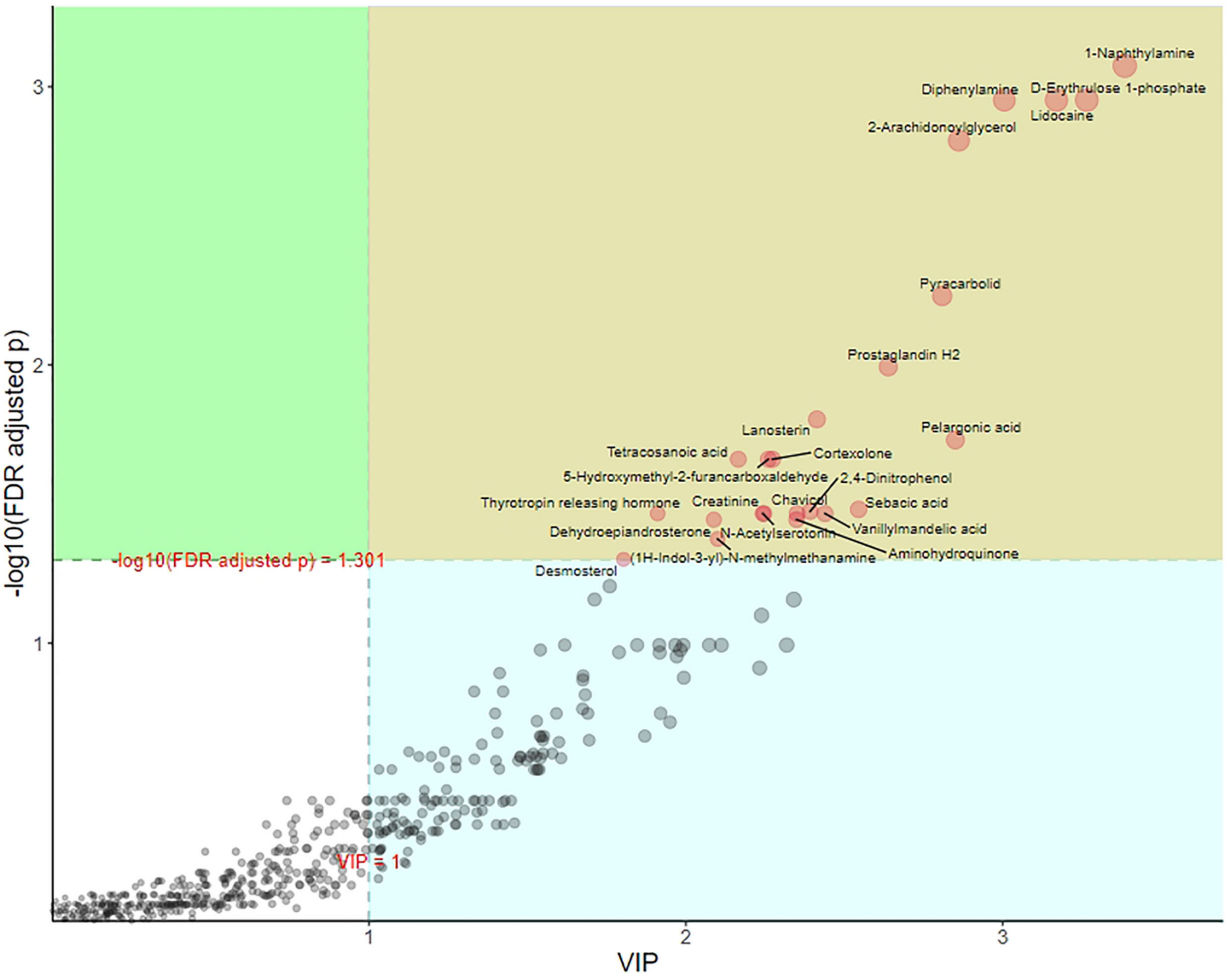
OPLS-DA metabolite importance map of group of POR and controls. Each point represents a metabolite. The abscissa is VIP, and the ordinate is the p value after FDR correction (log10 transformation).

The process of discriminant analysis is used to find the metabolites that play the most important role (value importance projection, VIP) and can be used as biomarkers to distinguish different groups. As shown in **Figure 6**, the metabolites marked with the name in the yellow area were those with *p <*0.05 and VIP >1. These metabolites had significant differences between groups, which play an important role in OPLS-DA and should be paid attention to.

**Figure 7:**
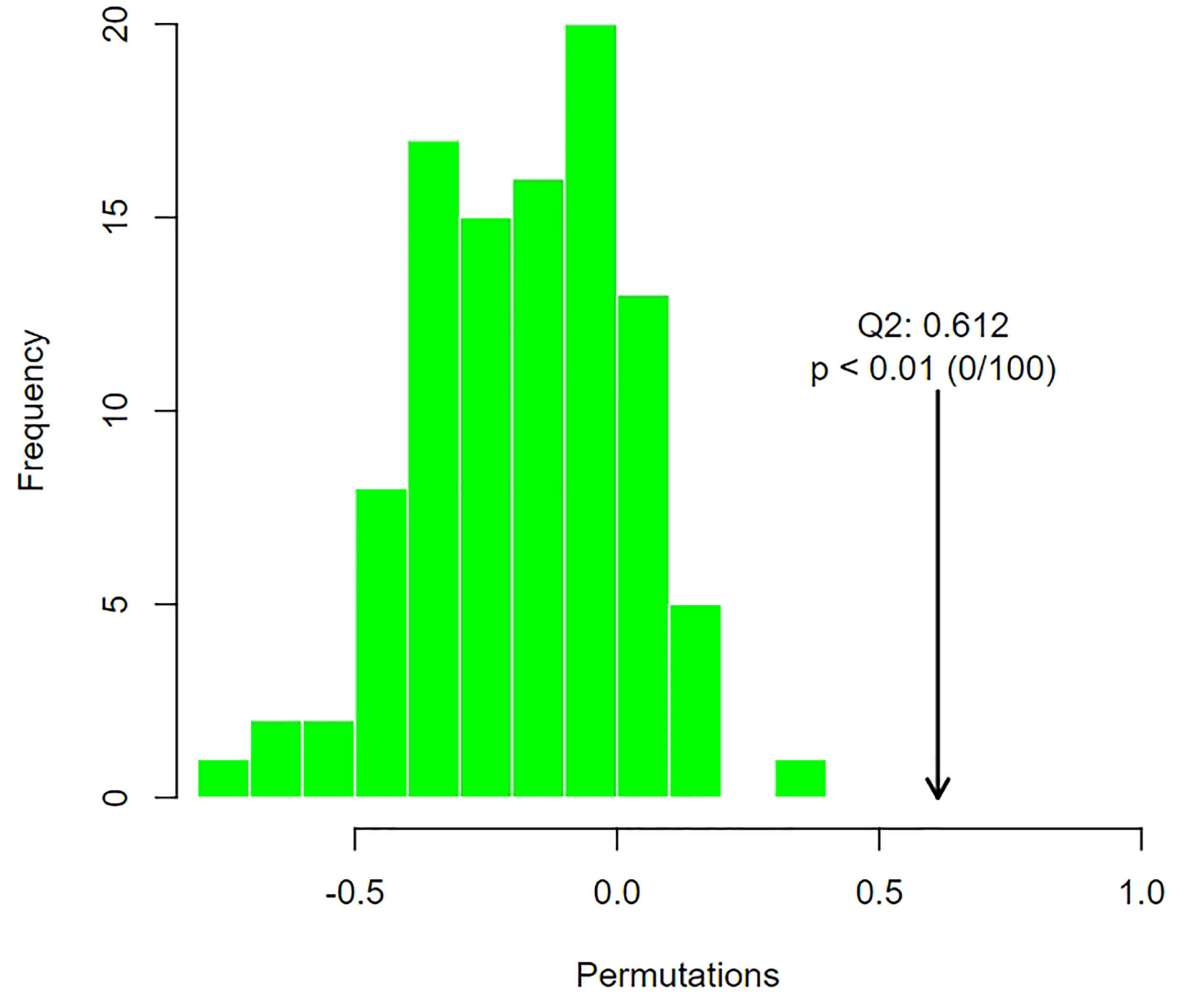
Distribution of test statistics (Q^2^) and P value of OPLS-DA permutation test of group of POR and controls. The distribution diagram is the permutation random distribution of Q^2^, and the arrow refers to the actually observed model Q^2^.

In the permutation test of OPLS-DA, we used Q^2^ as the test statistic and used the permutation method to obtain the random distribution of Q^2^ to confirm the stability and robustness of the model. As shown in **Figure 7**, the observed Q^2^ value (Q^2^ = 0.612) was significantly greater than the random value, so the prediction ability of the model was significant and there should be metabolites with significant differences between groups.

### Univariate Analysis of Metabolite Differences

**Figure 8:**
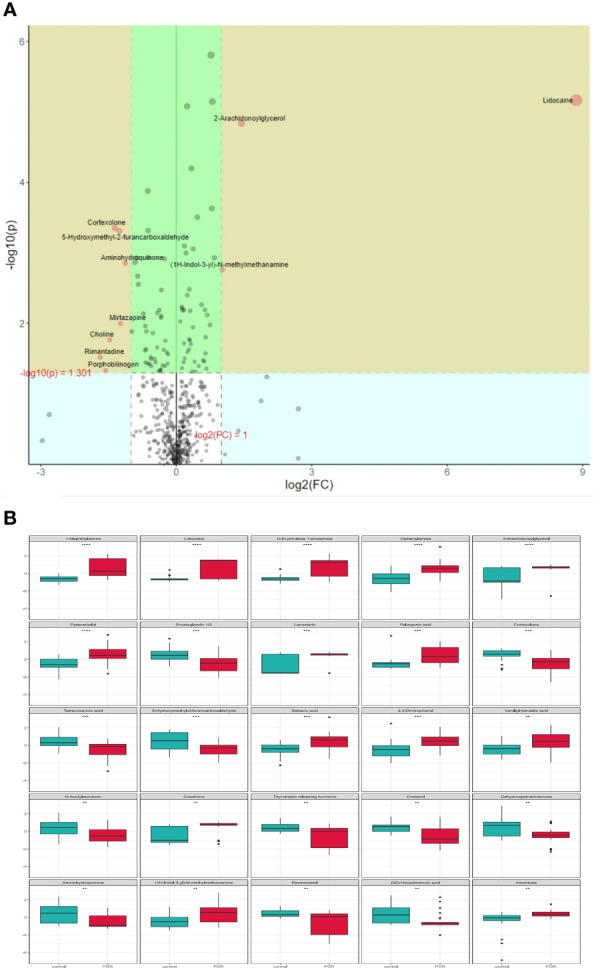
Volcano features **(A)** and box diagram **(B)** of metabolite differences. **p < 0.01, ***p < 0.001, ****p < 0.0001.

Assessing the impact of changes in metabolites on organisms is necessary, not only knowing whether the changes exist. We can measure the changes by calculating the fold change (FC) of metabolites, and select some important metabolites by combining *p* values. The increase multiple is positive and the decrease multiple is negative. As shown in **Figure 8A**, the metabolites in the yellow area of volcano features were those with *p <*0.05 and FC >2. These metabolites had significant differences between groups, which should be paid attention to.

In order to intuitively show the differences of metabolites between groups, we made a box chart of the top representative metabolites (the top 25 with smaller *p* value) (**Figure 8B**). Lidocaine, 1-naphthylamine, D-erythrulose1-phosphate, diphenylamine, 2-arachidonoylglycerol, pyracarbolid, lanosterin, pelargonic acid, sebacic acid, and 2,4-dinitrophenol were upregulated in the POR group compared with the control group (*p <*0.001). Prostaglandin H2, 5-hydroxymethyl-2-furancarboxaldehyde, cortexolone, and tetracosanoic acid were downregulated in the POR group compared with the control group (*p* < 0.01).

### Random Forest Analysis and Support Vector Machines

**Figure 9:**
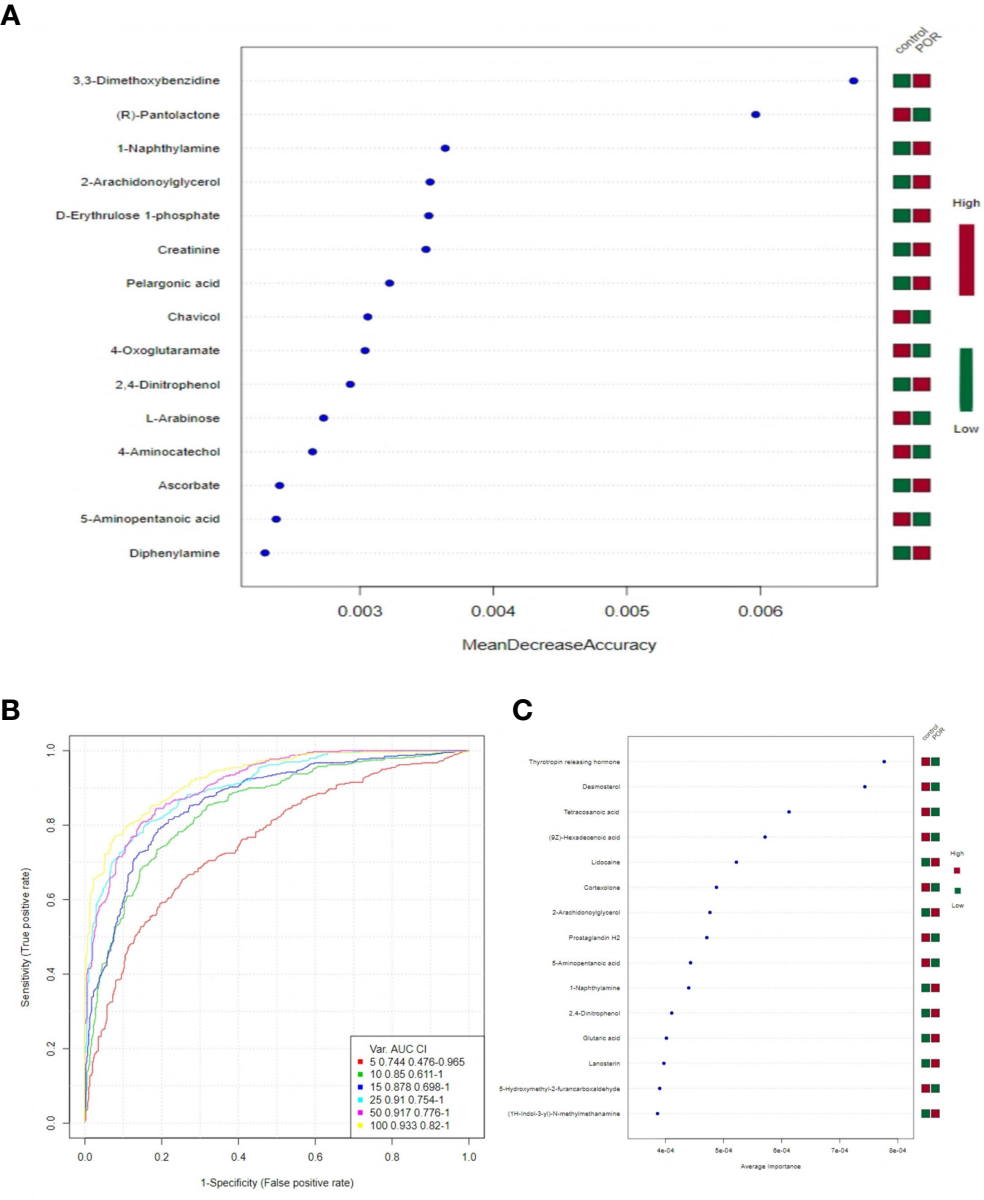
15 most important metabolites in random forest **(A)**, SVM ROC curve **(B)**, the 15 most important metabolites in SVM **(C)**. **(A)** The abscissa on the left is “mean decrease accuracy”, which measures the importance of a metabolite in random forest; on the right is a thermogram of the contents of the 15 metabolites in the two groups. **(B)** The abscissa is specificity and the ordinate is sensitivity. **(C)** The abscissa on the left is the average importance of SVM, and the heatmap on the right is the content of the 15 metabolites in the two groups.

In random forest analysis, we used “mean decrease accuracy” and “mean decrease gini” to measure the importance of a metabolite. If the value of a metabolite was changed into a random number, the decrease degree of prediction accuracy of random forest was “mean decrease accuracy”; “mean decrease gini” was the effect of a metabolite on the heterogeneity of observations at all nodes of the classification tree. The larger the two values were, the more important the metabolites were in the random forest. **Figure 9A** shows the 15 most important metabolites in the random forest, which were quite different between groups.

The closer the area under the curve (AUC) was to 1, the higher the accuracy and specificity was, and the better the predicted effect of the model (21). **Figure 9B** shows that the better predictions of the model and the greater differences of metabolites were between groups.

Combining the bagging method with SVM, we listed the 15 most important metabolites in SVM, which were significantly different between groups (**Figure 9C**). They were the thyrotropin releasing hormone, desmosterol, tetracosanoic acid, lidocaine, 2-arachidonoylglycerol, 9Z-hexadecenoic acid, cortexolone, prostaglandin H2, 1-naphthylamine, 5-aminopentanoic acid, 5-hydroxymethyl-2-furancarboxaldehyde, D-erythrulose1-phosphate, 1H-indol-3-yl-N-methylmethanamine, lanosterin, and 2,4-dinitrophenol.

### Topological Pathway Enrichment of Metabolites

**Figure 10:**
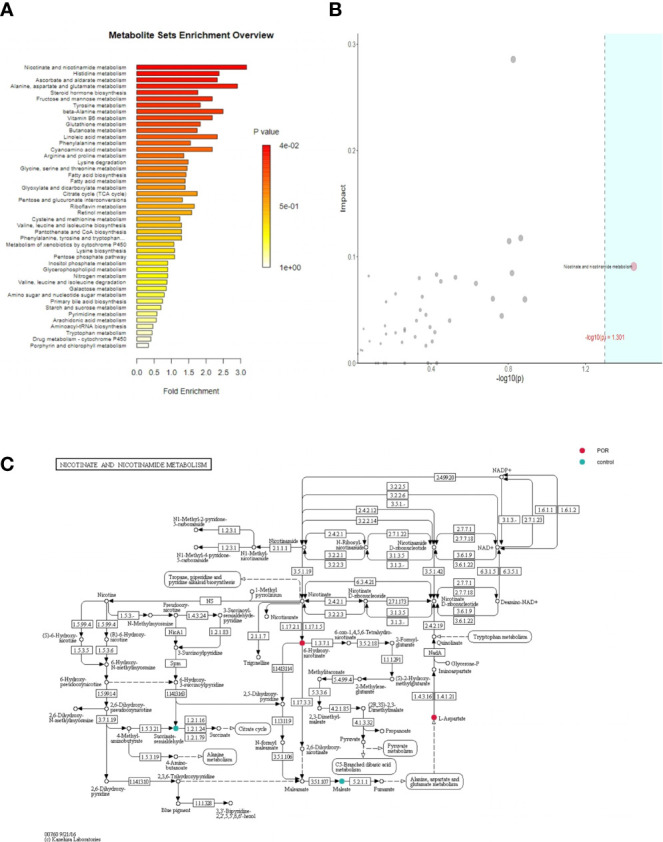
Enrichment analysis of over-representation analysis (ORA) **(A)**, topoenrichment analysis **(B)**, and the metabolic pathway gene map **(C)**. **(A)** The abscissa is enrichment factor, which is the number of observed metabolites/theoretical metabolites in metabolic pathway. The size of P value is expressed by color. The darker the color is, the smaller the p value is. **(B)** The abscissa is the p value of ORA analysis, and the blue area is significant (P < 0.05); The ordinate is the topological analysis impact. **(C)** Colored metabolites are metabolites with significant differences between groups. Color corresponds to the group, indicating that the content of metabolites in the corresponding group is higher (compared with the other group).

The purpose of enrichment analysis is to find the biological pathway which plays a key role in a biological process, so as to reveal and understand the basic molecular mechanism of the biological process (22). **Figure 10A** shows the metabolic pathway of significant enrichment of different metabolites (*p <*0.05), which might be of great significance in POR. We listed six metabolic pathways with significant enrichment, which were nicotinate and nicotinamide metabolism, steroid hormone biosynthesis, tyrosine metabolism, vitamin B6 metabolism, butanoate metabolism, and phenylalanine metabolism.

Topological analysis calculated the effect of significant metabolites in metabolic pathways (measured by the impact). **Figure 10B** shows that the metabolic pathways in the blue region were significant in the over-representation analysis (ORA) enrichment analysis, and the ordinate showed the impact of nicotinate and nicotinamide metabolism in the topological analysis.

**Figure 10C** shows the metabolic pathway gene map. The color corresponds to the group, indicating that the content of metabolites in the corresponding group is significantly higher (compared with the other group). In the nicotinate and nicotinamide metabolism pathway, L-aspartic acid and 6-hydroxynicotinate in the POR group were significantly higher than in the control group, while maleic acid and succinic acid semialdehyde in the POR group were significantly lower than in the control group.

## Discussion

Metabolites refer to the substances produced or consumed through metabolic processes, excluding biological macromolecules. Theoretically, metabolites should include nucleic acids, proteins, lipids, and other small molecular metabolites. The metabolome refers to the dynamic whole of endogenous metabolites. Metabolomics is a new branch of science that qualitatively and quantitatively analyzes all low molecular weight metabolites of an organism or cell in a specific physiological period (20). Metabolomics research can be divided into untargeted and targeted. Untargeted metabolomics can detect all detectable metabolite molecules in the sample without bias, and conduct difference analysis and pathway analysis through the bioinformatics method to find metabolomic biomarkers (23).

POR is a cause of infertility, and it infers a low natural pregnancy rate and poor pregnancy outcome of *in vitro* fertilization (IVF). Predictive markers and appropriate pretreatment and treatment plans of POR may help clinicians improve the pregnancy rate in a short time and at a low cost (24). Researchers have made many efforts in this area, such as the formulation of the Poseidon classification (25), which comprehensively considered the age, the ovarian reserve markers (AMH and AFC), and responsiveness of POR patients.

In this study, we investigated the relationship between metabolites and physiological and pathological changes by using untargeted metabolomics to study the differences of metabolite expression, providing some potential biomarkers and metabolic pathways for the prediction of POR.

In our study, the clinical characteristics of 25 POR women and 25 control women with matched age had statistically significant differences in the levels of FSH, AMH, and AFC (*p <*0.001), which was consistent with other studies (26, 27). The differences in age, LH, E_2_, and T in both groups were not significant (*p >*0.05). PRL in the POR group was significantly lower than in the control group (*p <*0.05), which was little analyzed in another POR study (28). There are many influencing factors in PRL, which may have no clinical significance in the prediction markers of POR.

In our study, after the QC and standardization of plasma sample data, we identified 538 metabolites with significant differences in the two groups. Our results showed that POR had significantly higher expressions of 1-naphthylamine, lidocaine, D-erythrulose1-phosphate, diphenylamine, 2-arachidonoylglycerol, pyracarbolid, lanosterin, pelargonic acid, sebacic acid, and 2,4-dinitrophenol than the control group (*p <*0.001). POR had significantly lower expressions of prostaglandin H2, cortexolone, tetracosanoic acid, and 5-hydroxymethyl-2-furancarboxaldehyde than the control group (*p <*0.01). Combining univariate analysis and the bagging method with SVM, we listed the nine most important metabolites with significant differences (**Figure 9C**), which were tetracosanoic acid, 2-arachidonoylglycerol, lidocaine, cortexolone, prostaglandin H2, 1-naphthylamine, 5-hydroxymethyl-2-furancarboxaldehyde, D-erythrulose1-phosphate, and 2,4-dinitrophenol (*p <*0.05). Combining metabolomic profiling together with SVM and pathway analysis, the nicotinate and nicotinamide metabolism pathway, including L-aspartic acid, 6-hydroxynicotinate, maleic acid, and succinic acid semialdehyde, was identified to have a significant difference in POR women compared to control women (*p <*0.05), which may be associated with ovarian reserve.

It can be seen from the metabolic network diagram that the nicotinate and nicotinamide metabolism pathway is mainly related to ovarian reserve.

Nicotinate and nicotinamide metabolism has been widely studied in many fields, such as disease treatments and stem cell applications. Nicotinic acid and nicotinamide are two forms of water-soluble B vitamin B3, also known as vitamin PP. As the basic components of coenzymes, they participate in body synthesis and catabolism, and have important effects on animal carbohydrate, lipid, and protein metabolism, and regulate oxidative stress (29). Nicotinamide riboside (NR) treatment in aging mice can delay the degeneration of muscle, enhance muscle function, and increase mouse life span (30). Nicotinamide, as an inhibitor of multiple kinases, can affect the pluripotency and differentiation of human embryonic stem cells (31). Nicotinic acid has long been believed to have a favorable effect on plasma lipids, lowering plasma low density lipoprotein (LDL) and raising high density lipoprotein (HDL) (32). NR supplementation can enhance oxidative metabolism by activating SIRT1 and SIRT3 in high-fat diet-induced mammalian cells and mouse tissues, and improve metabolic abnormalities (33). Excess niacin consumption may cause increased appetite, involve oxidative stress, and increase the prevalence of obesity in US children (34).

Nicotinate and nicotinamide metabolism has also been studied in obstetrics and gynecology. One animal study showed different concentrations of niacin in the medium could impact the maturation quality of *in vitro* embryo production (IVP) embryos and total tolerance of bovine oocytes to vitrification (35). Polycystic ovary syndrome (PCOS) is one of the common reproductive endocrine diseases for fertility in women of reproductive age. The clinical manifestations of PCOS are highly heterogeneous, including sparse ovulation or prolonged anovulation, hyperandrogenemia, insulin resistance, infertility, dyslipidemia, and obesity. For PCOS, additional supplementation of B1, folates with inositol, niacinamide, and antioxidants in the diet should be recommended to improve insulin sensitivity (36). Nicotinamide might be partially mediated in an adenosine 5’-monophosphate (AMP)-activated protein kinase (AMPK) manner, and was found to reduce the serum testosterone levels and CYP17A1 gene expression in a letrozole-induced rat model of PCOS (37).

Administration of niacin promoted follicle growth by increasing germ-line cell marker DDX4 and cell proliferation marker PCNA in the ovary, inhibited apoptosis *in vitro*, and treated premature ovarian failure (POF) in mouse models, which were under harmful conditions, such as radiation and chemotherapy damage (38). It was reported that consistent administration of nicotinamide with appropriate concentrations could maintain DNA integrity and prevent the progression of programmed cell death (PCD) (39). Nicotinamide as a dietary supplement might protect cells against apoptosis induced by sodium deoxycholate (NaDOC), independent of oxidative stress (40). Imidacloprid (IMI) is a worldwide highest selling insecticide, which is more toxic to the mammalian nervous system. 6-hydroxynicotinic acid, as one of the major metabolites of IMI, was observed in female rats where the concentration was higher in the ovary and blood after oral administration of IMI (41). In this study, 6-hydroxynicotinic acid was higher in POR than controls, which may show that 6-hydroxynicotinic acid is toxic to the ovary. POR women may contact more IMI or other hazardous substances and environmental exposure than controls. N1-methylnicotinamide was reported to postpone aging based on the activation of the ovarian AMPK in a rat model of PCOS (42). Some metabolisms in the nicotinate and nicotinamide metabolism pathway may play an important role in POR.

## Conclusion

In our present study, the metabolic alterations in POR and normal patients were characterized using an LC–MS-based metabolomics approach. Clear metabolic differences were observed between POR and controls. These variations involved significant perturbations in glycerophospholipid, glycerolipid, and fatty acid metabolisms. The nicotinate and nicotinamide metabolism pathway may offer new insight for complementary prediction and treatment of POR. It is important to pre-assess the ovary function of women of childbearing age and adopt an individualized scheme for their family planning. However, this study had some limitations as well. The sample size included was small, and the research results may be biased to some extent. The functional associations of this metabolomics in POR need further investigation. Our findings also suggest that LC–MS-based metabolomics research offers a promising strategy for the complementary diagnosis of POR. The counterbalance of the corresponding molecular events might contribute to the prediction, alleviation, and treatment of POR.

## Strengths and Limitations

Our results demonstrated that the nicotinate and nicotinamide metabolism pathway had an important effect in POR using the untargeted LC-MS metabonomic method. There were some limitations in this research including the small sample size and the lack of an underlying mechanism. In the future, first, we should plan to conduct more research with a sufficient number of participants. Next, we should conduct cell experiments and animal experiments to verify the effect and pathogenesis of this pathway in POR.

## Data Availability Statement

The original contributions presented in the study are included in the article/supplementary materials. Further inquiries can be directed to the corresponding authors.

## Ethics Statement

The studies involving human participants were reviewed and approved by the Ethics Committee of Shanxi Provincial People’s Hospital. The patients/participants provided their written informed consent to participate in this study. Written informed consent was obtained from the individual(s) for the publication of any potentially identifiable images or data included in this article.

## Author Contributions

HS designed the main study. CY, LF, and FZ contributed to data collection. HS and HL performed the statistical analysis and interpreted the results. HS wrote the manuscript. QQ contributed to the critical revision of the article. All authors reviewed and approved the final version.

## Funding

The National Natural Science Foundation of China (81401192) supported QQ for this study.

## Conflict of Interest

The authors declare that the research was conducted in the absence of any commercial or financial relationships that could be construed as a potential conflict of interest.

## Publisher’s Note

All claims expressed in this article are solely those of the authors and do not necessarily represent those of their affiliated organizations, or those of the publisher, the editors and the reviewers. Any product that may be evaluated in this article, or claim that may be made by its manufacturer, is not guaranteed or endorsed by the publisher.
